# Hypertension, mitral valve disease, atrial fibrillation and low education level predict delirium and worst outcome after cardiac surgery in older adults

**DOI:** 10.1186/s12871-018-0481-0

**Published:** 2018-02-01

**Authors:** Fátima R. Oliveira, Victor H. Oliveira, Ítalo M. Oliveira, José W. Lima, Daniela Calderaro, Danielle M. Gualandro, Bruno Caramelli

**Affiliations:** 1 Heart Institute (InCor), University of Sao Paulo, Brazil. Av. Dr. Enéas Carvalho de Aguiar 44, Sao Paulo, CEP, São Paulo, 05403-000 Brazil; 2 Universidade Estadual do Ceará, Fortaleza, Brazil; 3 Hospital de Messejana Dr. Carlos Alberto Studart Gomes SESA, Fortaleza, Ceará Brazil

**Keywords:** Delirium, Cardiac surgery, Incidence, Elderly, Illiterate

## Abstract

**Background:**

Delirium is a common complication after cardiac surgery in older adult patients. However, risk factors and the influence of delirium on patient outcomes are not well established. We aimed to determine the incidence, predisposing and triggering factors of delirium following cardiac surgery.

**Methods:**

One hundred seventy-three consecutive patients aged ≥60 years were studied. Patients’ characteristics and two cognitive function assessment tests were recorded preoperatively. Perioperative variables were blood transfusion, orotracheal intubation time (OIT), renal dysfunction, and hypoxemia. Delirium was assessed using the Confusion Assessment Method for the Intensive Care Unit. The composite outcome consisted of death, infection, and perioperative myocardial infarction until hospital discharge or 30 days after surgery, and for up to 18 months.

**Results:**

One hundred six patients (61.27%) were men and the age was 69.5 ± 5.8 years. EuroSCORE II index was 4.06 ± 3.86. Hypertension was present in 75.14%, diabetes in 39.88%, and 30.06% were illiterate. Delirium occurred in 59 patients (34.1%). Education level (OR 0.81, 0.71–0.92), hypertension (OR 2.73, 1.16–6.40), and mitral valve disease (OR 2.93, 1.32–6.50) were independent predisposing factors for delirium, and atrial fibrillation after surgery (OR 2.49, 1.20–5.20) represented the potential triggering factor**.** Delirium (OR 2.35, 1.20–4.58) and OIT ≥ 900 min (OR 2.50; 1.30–4.80) were independently associated with the composite outcome.

**Conclusions:**

In older adult patients submitted to cardiac surgery, delirium is a frequent complication that is associated with worst outcome. Independent risk factors for delirium included education level, hypertension, mitral valve disease, and atrial fibrillation after cardiac surgery.

## Background

Delirium is a transient and fluctuating course condition of acute onset that is characterized by reduced level of consciousness, global cognitive dysfunction, and disorder in the sleep-wake cycle. In patients undergoing cardiac surgery, delirium is a complication that affects 2–57% of the patients, reaching as high as 73% in older subjects [1].

Delirium has a multifactorial etiology. Several risk factors for delirium after cardiac surgery have been described in the literature as age, depression, anemia, atrial fibrillation before intervention as predisposing factors; and orotracheal intubation time (OIT), extracorporeal circulation time, hypoxemia as precipitating factors [2–5]. It seems that the complex interaction between these factors is responsible for the development of delirium. Low educational level has also been studied as a risk factor for delirium after surgery but never in a group of very low educational level that includes a significant rate of illiteracy.

Postoperative delirium is strongly associated with higher morbidity and mortality [6]. The identification of patients at high risk for delirium is important to deliver proper care and avoid the consequences of this complication. The objective of this research was to determine the incidence, predisposing factors, and triggering factors of delirium following cardiac surgery and its consequences within 30 days of surgery and during a 12–18-month follow-up in older adult patients with a significant rate of illiteracy.

## Methods

### Population

This was an observational prospective cohort study at Messejana Hospital, a tertiary Hospital in Fortaleza, Brazil. Between September 2011 and December 2013 a group of 609 patients underwent cardiac surgery. Of these, 342 patients were aged ≥60 years, an inclusion criterion. The exclusion criteria included blindness, deaf-muteness, previous diagnosis of dementia confirmed by specialist, permanently bedridden due to sequel from a cerebrovascular accident, creatinine clearance < 30 mL/min or on dialysis, emergency cardiac surgery, surgery combined with vascular procedures such as carotid endarterectomy or aortic aneurysm repair, post-infarction inter-ventricular communication correction, isolated congenital heart disease, or the presence of delirium at the preoperative interview. Local ethical committee (CAPPesq) has approved the protocol under the number 825/11. Informed consent was obtained from all patients according to international guidelines.

### Clinical, surgical variables and outcomes

Demographic and clinical characteristics as well as the presence of comorbidities were obtained at preoperative evaluation. Preoperative tests included electrocardiogram, echocardiogram, carotid ultrasonography, coronariography, and cardiac troponin I. At this time, the cognitive function of patients was assessed using the mini mental state examination (MMSE) adjusted for education level according to Bertolucci’s classification [7], and the verbal fluency test, including words from the animal category (VFT), also adjusted for education level [8].

For evaluation of preoperative delirium, the confusion assessment method (CAM) was applied by two researchers, and diagnosis was determined by the presence of criteria 1 and 2 plus either criteria 3 or 4 [9]. Data related to the intervention were also recorded: duration of surgery and anesthesia, cardiopulmonary bypass or aortic clamping time (when used), and transfusion of blood components.

Postoperative variables included hematocrit and hemoglobin values obtained immediately after patient’s arrival in the intensive care unit (ICU), OIT, and date of hospital discharge and/or death. To detect postoperative delirium, patients were observed daily from the first postoperative day in the ICU until hospital discharge. Owing to its ability to be used in mechanically ventilated patients, the CAM-ICU tool [10] was performed during ICU stay. The diagnosis was confirmed according to the criteria of the Diagnostic and Statistical Manual of Mental Disorders (DSM-IV) [11]. The diagnosis of hypoxemia required a ratio of PaO2/FIO 2 < 200 analyzed at least once daily in the immediate postoperative and in the third day thereafter in patients on mechanical ventilation or breathing spontaneously, and renal dysfunction was determined using RIFLE the criteria [12].

The consequences of delirium were evaluated up to 30 days after surgery using the composite endpoint, consisting of death from all causes, infections, and perioperative acute myocardial infarction (AMI). Infection was defined based on criteria established by National guidelines for health care-related infections [13]. Only serious nosocomial infections requiring treatment with intravenous antibiotics during the same hospital stay were captured.

For the diagnosis of perioperative AMI, electrocardiographic criteria and troponin I (> 10 times the 99th percentile of the upper reference limit) were used as markers of myocardial injury, measured in the first, third, and fifth postoperative day.

Patients were followed up for 12 to 18 months at the outpatient clinic and were reassessed for diagnosis of dementia using MMSE and VFT.

### Statistical analysis of data

Descriptive statistics such as means, standard deviations, frequencies and percentages were computed. Continuous variable distributions were compared using t-test for independent or paired samples, or Mann-Whitney U tests, as appropriate. Proportions were compared using Pearson’s Chi Squared or Fisher’s Exact tests. The relationship between variables and delirium or composite outcome was assessed through simple logistic regression.

Initially, reported variables associated to delirium described in the literature [4], and variables associated to delirium with *p*-value ≤0.1 in the present study, were eligible for the multivariate analysis. Altogether, there were seventeen potential predictor variables. Taking in account the number of events of delirium in the study sample (59 events), and the number of variables in a multivariate model, which would include seventeen predictor variables [14], we decided to use a stepwise forward selection, with backward elimination modeling strategy [15]. Model building was processed with forward variable selection and backward variable deletion. Initially, we developed a reference model with the intercept followed by simple regression models, one for each independent variable. Each independent variable model was compared with the reference model through the Likelihood Ratio Test (LRT). The independent variable with the smallest *P*-value, smaller than 0.2 (entry criteria), was the first variable selected. This simple logistic model was used for the next step of variable selection. This process was repeated until the independent variable with the smallest *p*-value was equal or greater than 0.2. When a new variable was included in a model, logistic models excluding one independent variable, one at a time, were compared with the full model. The independent variable with the highest LRT p-value greater than 0.4 was excluded (deletion criteria). The same procedures described before were adopted to build a model of the composite outcome. MMSE and the VFT were evaluated as continuous variables and the values were compared before surgery and after 1 year of their completion.

Kaplan-Meier curves were compared by means of Log-Rank Test for equality of survivor functions. All statistics tests were two-tailed and a *P*-value smaller than 0.05 was accepted as statistically significant.

## Results

### General characteristics of the patients in the study

**Fig. 1:**
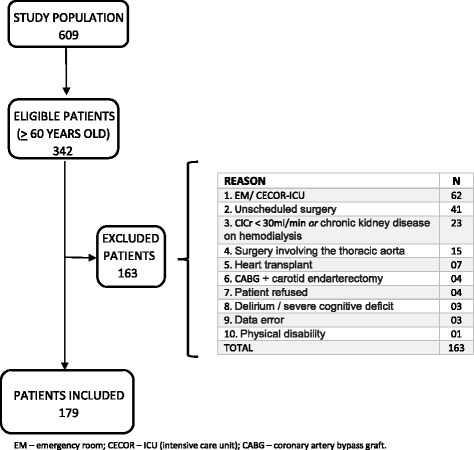
Study population flowchart

After exclusion criteria, 179 were included in the study. Final sample was reduced to 173 patients as the presence of delirium could not be evaluated in six. Flow chart showing the process of including patients in the study is depicted in Fig. 1. General characteristics of the study population is shown in Table 1. A total of 148 (85.5%) patients were using beta-blockers, 152 (87.6%) were taking statins, 120 (69.3%) were using aspirin the day before surgery, and only eleven (6.4%) were using antidepressants. The level of education was low considering the average of 3.05 ± 3.08 years and illiteracy rate of 30.1%. In 109 (63.0%) patients, a coronary angiography revealed three-vessel or left main coronary artery lesions and thirty-two (18.5%) had no significant coronary disease. Forty-three (24.9%) patients had mitral valve disease, and thirty (17.3%) had aortic valve disease detected by Doppler echocardiography. The ultrasound of carotid and vertebral arteries revealed obstructive lesions ≥70% in 10 patients (5.8%), and 79 (46.2%) showed no obstruction. Of the 173 patients, 114 (65.9%) underwent isolated coronary artery bypass graft (CABG), eighteen (10.4%) had aortic prosthesis implantation, twenty-one (12.1%) received mitral prosthesis, and four (2.3%) had both mitral and aortic prosthesis implants. Finally, in sixteen (9.2%) patients, CABG was associated with repair and/or implant of a prosthetic valve.

**Table 1:** Baseline clinical characteristics of the patients in the study (*n* = 173)

Variables	Values
Age^a^	69.50 ± 5.80
Male	106 (61.3%)
Body mass índex^a^	26.20 ± 4.10
Level of education (years)	3.05 ± 3.08
Illiterate	52 (30.1%)
Systemic arterial hypertension	130 (75.1%)
Diabetes mellitus	69 (39.9%)
Dyslipidemia	123 (71.1%)
Previous stroke	27 (15.6%)
Smoking	
Non smoker	123 (71.1%)
Current smoker	30 (17.3%)
Ex-smoker	20 (11.6%)
Alcohol consumption	34 (19.6%)
EuroSCORE II^a^	4.06 ± 3.86
Ejection fraction (ECO)^a^	0.53 ± 0.12
Cardiac rhythm (sinus rhythm)	153 (88.4%)
Pre MMSE^a^	23.27 ± 4.51
Pre VFT^a^	10.31 ± 3.75
Pre ClCr^a^	60.73 ± 19.74

*ECO* Doppler echocardiography, *MMSE* Mini-mental state examination, *VFT* Verbal fluency test, *ClCr* Creatinine clearance

^a^Continuous variables are described as means and standard deviation. Other variables are described as absolute and percentage values

The incidence of delirium was 34.1%; in 41 (69.5%) it was detected in the first two postoperative days, in nine (15.2%) on the third day, in six (10.2%) on the fourth day, and in three (5.1%) on the fifth day. Hyperactive delirium was found in seventeen (28.8%) patients, hypoactive delirium in 24 (40.7%), and mixed delirium in eighteen (30.5%). The overall mortality rate was 12.1%. In-hospital death occurred in ten (5.8%) patients, and eleven (6.3%) died after hospital discharge. Of these, 9 (42,8%) had postoperative delirium and 12 (57,2%) did not.

### Perioperative variables and their relationship with delirium

Age, low educational level, mitral valve disease, preoperative MMSE and VFT, aortic clamping time, postoperative renal dysfunction, atrial fibrillation detected after cardiac surgery and troponin I measured in the first day post-surgery were the risk factors identified in univariate analysis Table 2.

**Table 2:** Pre, trans, and post-operative variables and their relationship with delirium

Variable	Delirium		*p*
	No (*n* = 114)	Yes (*n* = 59)	
Preoperative variables			
Male	75 (65.79%)	31 (52.54%)	0.09
Age	68.80 ± 5.63	70.80 ± 6.05	0.03
Education level (years)	3.59 ± 3.19	2.00 ± 2.55	< 0.01
Hypertension	81 (71.05%)	49 (83.05%)	0.08
Statin use	104 (91,2%)	48 (81.35%)	0.06
EuroSCORE II	3.65 ± 3.91	4.83 ± 3.65	0.06
Valve disease			
Mitral valve disease	22 (19.29%)	21 (35.59%)	< 0.01
Aortic valve disease	18 (15.78%)	12 (20.33%)	0.14
Pre MMSE	24.04 ± 4.15	21.79 ± 4.85	< 0.01
Pre VFT	10.92 ± 3.88	9.11 ± 3.21	< 0.01
ClCr (mL/min)	62.60 ± 19.80	57.00 ± 19.10	0.07
Trans/post-operative variables			
Anesthesia time (min)	274.10 ± 60.62	290.10 ± 57.11	0.09
CPB time (min)	64.99 ± 52.83	81.55 ± 59.90	0.06
Aortic clamping time (min)	42.83 ± 38.90	58.88 ± 46.98	0.02
Hypoxemia	36 (31.58%)	27 (45.76%)	0.06
Renal dysfunction	19 (16.66%)	18 (30.51%)	0.03
AF post-surgery	26 (22.8%)	28 (47.45%)	< 0.01
Troponin I [1]	6.62 ± 8.84	12.84 ± 17.47	< 0.01

*MMSE* Mini-mental state examination, *VFT* Verbal fluency test, *ClCr* Creatinine clearance, *CPB time* Cardiopulmonary bypass time, *AF* Atrial fibrillation; Troponin I [1]: troponin dosed in the first day after surgery

MMSE adjusted according to the patient’s education level revealed that only thirteen (7.5%) patients had preoperative cognitive deficit; in five (38.5%) of these, the diagnosis of delirium was made after surgery. We observed no association between delirium and the pre-existence of cognitive deficit. Among those who did not have previous cognitive deficit, delirium was present after intervention in 54 (33.7%). However, there was a significant association of low education level with delirium Table 2.

Similarly, VFT results adjusted by education level detected 70 (40.5%) patients with cognitive deficit. Of these, twenty-eight (40%) had delirium. In those patients without cognitive deficit by the VFT, delirium occurred in thirty-one (30.1%). However, we observed no association of education-adjusted VFT and the occurrence of delirium.

The preoperative variables listed in Table 2 were used to identify the predisposing factors for delirium, while perioperative variables covered the model identifying triggering factors in the multivariate analysis. Data are shown in Table 3.

**Table 3:** Multivariate model of risk factors for delirium after cardiac surgery

Variable	OR	Standard error	95% CI	*p* value
Predisposing factors				
Education level (years)	0.81	0.05	0.71–0.92	< 0.01
Systemic arterial hypertension	2.73	1.18	1.16–6.40	0.02
Valve disease				
Mitral valve disease	2.93	1.19	1.32–6.50	< 0.01
Aortic valve disease	1.55	0.71	0.63–3.84	0.33
Triggering factors				
AF post-surgery	2.49	0.93	1.20–5.20	0.01

*OR* Odds ratio, *95% CI* 95% confidence interval, *AF* Atrial fibrillation

### *Delirium* as a risk factor for the composite outcome and clinical follow-up

Univariate analysis showed that delirium, OIT time ≥ 900 min and time in the intensive care unit were associated to the composite outcome (death, postoperative infection and perioperative myocardial infarction). Results are depicted on Table 4. Multiple logistic regression model was created with the variables listed in Table 4 to identify significant factors for the occurrence of the composite outcome. Data are shown in Table 5.

**Table 4:** Pre, trans, and postoperative variables and their relationship to the composite outcome

Variable	OR	95% CI	*p* value
Male	0.59	0.32–1.11	0.10
Age	1.04	0.99–1.10	0.07
Diabetes mellitus	1.54	0.82–2.87	0.17
Carotid ultrasonography	1.63	0.85–3.11	0.13
Length of stay in ICU	1.22	1.10–1.36	< 0.01
Renal dysfunction postoperative	1.93	0.92–4.03	0.07
Delirium	2.67	1.39–5.11	< 0.01
OTIT ≥ 900 min	2.79	1.50–5.20	< 0.01
Postoperative AF	1.76	0.91–3.39	0.08

*OR* Crude odds ratio, *95% CI* 95% confidence interval, *ICU* Intensive care unit, *OTIT* Orotracheal intubation time, *AF* Atrial fibrillation

**Table 5:** Multivariate model of predictor factors to the composite outcome (death, postoperative infection and perioperative myocardial infarction)

Variable	OR	Standard error	95% CI	*p* value
Delirium	2.35	0.80	1.20–4.58	0.01
OTIT ≥ 900 min	2.50	0.83	1.30–4.80	< 0.01

*OR* Odds ratio, *95% CI* 95% confidence interval, *OTIT* Orotracheal intubation time as a category variable

**Fig. 2:**
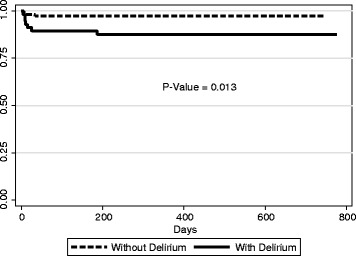
Time to death Kaplan-Meier Curves for patients with and without delirium

Of the 173 patients, 144 completed the follow-up. Comparison of data from patients before and 12–18 months after surgery, showed no differences between those with and without delirium: MMSE (24.0 ± 4.1 and 21.8 ± 4.8, before, 24.5 ± 3.6 and 22.4 ± 4.4, after 12–18 months, respectively, *P* = 0.38) and VFT (10.9 ± 3.9 and 9.1 ± 3.2, before, 12.4 ± 3.7 and 10.9 ± 4.1, after 12–18 months, respectively, *P* = 0.51). Thirty patients were readmitted after performing heart surgery, leading to a re-hospitalization rate of 20%. Of these, nine (30%) had postoperative delirium after cardiac surgery. There was no association between delirium and readmission (*P* = 0.73). Importantly, there was significant association between delirium and in-hospital mortality (*P* = 0.03). Time do death Kaplan-Meier curve is depicted in Fig. 2. Conversely, no association was observed between delirium and post discharge mortality (*P* = 0.50).

## Discussion

The incidence of delirium in the present study is consistent with previous reports using similar variables such as age, type of surgery, and the CAM-ICU scale for assessing delirium [5]. A precipitant risk factor for the development of delirium described in the literature is the intensive care environment. In this study, all patients were admitted to an intensive care unit after cardiac surgery, but only 59 patients (34.1%) had delirium. In a recent study, researchers evaluated the relationship between the harmful environment of the ICU and the onset of delirium in postoperative cardiac surgery and found no significant association [16]. It is likely that the complex interaction between several risk factors favors the development of delirium and not the influence of a single one.

In the present study, low education level was an independent predictor of the occurrence of delirium. Considering that education level is frequently considered a proxy of cognitive reserve, we expected that the association between cognitive decline (comparing pre- and 12–18 months post-op test scores) and the occurrence of delirium would be significant. The association between education level and delirium has been previously investigated [17, 18] but data involving illiterate individuals submitted to cardiac surgery is lacking. Our findings can be explained by the fact that individuals who experience stimuli throughout life, such as literacy development in childhood and physical exercise, may delay the onset of dementia and its consequences [18]. Recently published work has revealed that postoperative delirium is associated with greater cognitive decline and a higher incidence of dementia up to 15 months after surgery [19]. Another study involving only with patients undergoing cardiac surgery revealed that postoperative delirium was associated with cognitive decline one month after surgery but this phenomenon disappeared when patients were re-evaluated after one year, suggesting possible recovery of cognitive function over time [20]. Literature data are still conflicting about the time of development of the cognitive deficit after postoperative delirium depending on the population studied, the length of follow-up, and the tools used for evaluation. [21–24]. We hypothesize that the relative short follow-up time of 12–18 months was not enough to detect a difference. In other words, in this 12–18 months follow-up study, having baseline low education level likely associated to low cognitive reserve and not cognitive decline could represent a factor of vulnerability of the brain and a risk factor for neurological disorders such as delirium or dementia.

The second independent risk factor for delirium identified in this study was hypertension. Previous research has shown that patients with hypertension are at a higher risk of developing delirium, after cardiac surgery [25, 26] or in the ICU [27–29]. The third independent risk factor for delirium in the present study was the presence of mitral valve disease. To date, there are no data in the literature showing this association. In our sample, there were 43 patients with mitral valve disease and 17 of them had also atrial fibrillation. We speculate that the simultaneous conditions like mitral valve disease, atrial fibrillation in patients with a large left atrium leading to microembolization phenomena could be responsible for the greater occurrence of delirium. These microemboli could lead to cerebral ischemia, and hypoperfusion of some regions of the brain that could represent the mechanism for the onset of delirium. Published studies have indeed revealed the presence of micro-emboli during cardiac surgery detected by imaging exams such as transcranial Doppler but the results remain contradictory [30, 31].

Atrial fibrillation after surgery was also identified as an independent precipitant risk factor for delirium after cardiac surgery in our sample. Recent studies have similar results [32–34]. The occurrence of atrial fibrillation postoperatively and its association with delirium favors the hypothesis of cerebral embolism and/or hypotension leading to a reduction of cerebral perfusion that can explain the pathophysiology of delirium [35, 36]. However, persistent atrial fibrillation preoperatively, in this study, was not a predictor of delirium after intervention.

On the other hand, we found that delirium was an independent risk factor for the composite outcome consisting of death, infection, and perioperative myocardial infarction. Several studies have previously identified delirium as a risk factor for increased morbidity and mortality during hospitalization, after one-year and over the long term [6, 37–40]. The cause and effect relationship between delirium and infection is not yet fully clear. Mechanisms have been proposed to explain brain dysfunction during sepsis, including disturbances in the production and release of neurotransmitters, changes in cerebral blood flow, and endothelial dysfunction [41]. We also found that OIT was an independent risk factor for the composite outcome. Indeed, ventilator-associated pneumonia (VAP) is the most common nosocomial infection in ICUs. Similarly, the mortality rate for patients with VAP can range from 24 to 50% [42].

### Study limitations

This research was carried out in a single center and therefore its results cannot be extrapolated to other centers or other populations. A tool for evaluating depression, common in the elderly patient, was not used, which may have some influence in the differential diagnosis between postoperative depression and hypoactive delirium difficult. Since delirium has fluctuating character throughout the day, it may have been underdiagnosed. In addition, the diagnosis of delirium was performed by experienced physicians in critical care medicine but not confirmed by neurologist or psychiatrist. The use of MMSE and VFT as sole evaluation tests of cognitive function may have underdiagnosed patients with Mild Cognitive Impairment (MCI). Finally, is a result of a study that includes the one-year follow-up of patients. We had to wait the one-year follow-up for all patients included in the study. This fact and the complexity of data involving several different aspects of clinical and surgical variables were the main reasons for the relative delay in publishing them.

## Conclusions

In summary, the incidence of delirium in older post-cardiac surgery adults was high in the studied population. The variables independently associated with delirium in this study were low education level, hypertension, and the presence of mitral valve disease as predisposing factors, and atrial fibrillation after cardiac surgery as a potential triggering factor. Delirium and prolonged oral intubation time (OIT ≥ 900 min) were independently associated with the composite outcome, consisting of all-cause mortality, perioperative myocardial infarction, and infection up to 30 days after surgery.

## Abbreviations

AMI: Acute myocardial infarction
CABG: Coronary artery bypass graft
CAM: Confusion assessment method
DSM-IV: Diagnostic and Statistical Manual of Mental Disorders
ICU: Intensive care unit
LRT: Likelihood Ratio Test
MCI: Mild Cognitive Impairment
MMSE: Mini mental state examination
OIT: Orotracheal intubation time
OR: Odds ratio
VFT: Verbal fluency test
